# Expression of aldehyde dehydrogenase 1 (ALDH1) is associated with basal-like markers and features of aggressive tumours in African breast cancer

**DOI:** 10.1038/sj.bjc.6605488

**Published:** 2009-12-15

**Authors:** H Nalwoga, J B Arnes, H Wabinga, L A Akslen

**Affiliations:** 1 Section for Pathology, The Gade Institute, University of Bergen, Haukeland University Hospital, N-5021, Bergen, Norway; 2 Department of Pathology, Makerere University College of Health Sciences, PO Box 7072, Kampala, Uganda

**Keywords:** ALDH1, BMI-1, basal-like, stem cells, African breast cancer

## Abstract

**Background:**

Putative breast cancer stem cells might express surface markers such as aldehyde dehydrogenase 1 (ALDH1) and BMI-1 proteins. The aim of this study was to explore the expression of these proteins in breast cancers from an African population and their associations with the basal-like phenotype (BLP) and other molecular characteristics.

**Methods:**

We analysed 192 paraffin-embedded breast carcinoma samples by tissue microarrays and immunohistochemical methods.

**Results:**

In total, 88 tumours (48%) expressed ALDH1, whereas 46 (25%) expressed BMI-1 protein. Expression of ALDH1 was associated with high histological grade (*P*<0.0005), high mitotic count (*P*<0.0005), high nuclear grade (*P*<0.0005), oestrogen receptor (ER) negativity (*P*<0.0005), progesterone receptor (PR) negativity (*P*=0.009), p53 expression (*P*=0.034), cytokeratin 5/6 positivity (*P*=0.008), epidermal growth factor receptor (EGFR) expression (*P*=0.015) and the BLP (*P*<0.0005), whereas it was inversely associated with BMI-1 staining (*P*=0.009). On the other hand, BMI-1 expression was associated with low histological grade (*P*=0.004) and ER positivity (*P*=0.001).

**Conclusion:**

There was a high prevalence of ALDH1 expression among breast carcinomas and associations with basal markers and features of aggressive tumours. Studies are required to elucidate the importance of these findings for improved understanding of breast cancer biology.

Human breast cancers have been reported to contain a sub-population of cancer cells similar to epithelial stem cells (Hamburger and Salmon, 1977; Al-Hajj *et al*, 2003; Abraham *et al*, 2005). These cells have the ability to self-renew and undergo differentiation to phenotypically diverse populations of tumour cells (Al-Hajj *et al*, 2003). It has been suggested that cancer stem cells drive the growth and spread of malignant tumours (Al-Hajj *et al*, 2003), and the stem cell hypothesis might have important implications for clinical management (Ginestier *et al*, 2007; Tanei *et al*, 2009).

The molecular diversity and subclassification of breast cancers have been reported in several studies during recent years (Perou *et al*, 2000; Sorlie *et al*, 2003; Rakha *et al*, 2008). Five tumour subgroups with different prognosis and response to adjuvant therapy have been identified. Of these, the basal-like and HER2 subtypes are of particular interest as both have a poor prognosis (Sorlie *et al*, 2001; Yang *et al*, 2007). The basal-like phenotype (BLP) is characterised by the expression of basal cell markers, and it overlaps with the triple-negative phenotype (TNP; ER−/PR−/HER2−) (Tischkowitz *et al*, 2007). It was reported that basal-like and *BRCA1*-associated breast carcinomas, which are also related (Foulkes *et al*, 2003) were both enriched with CD44+/CD24− candidate stem cells (Honeth *et al*, 2008), and *BRCA1* has been suggested to represent a stem cell regulator (Foulkes, 2004).

Previous studies indicate that stem cell-like populations in breast tissue are characterised by the expression of aldehyde dehydrogenase 1 (ALDH1), and breast cancer stem cells were isolated on the basis of increased ALDH1 expression (Ginestier *et al*, 2007). Thus, in the breast, expression of ALDH1 is considered to be a marker of both normal and malignant stem and progenitor cells (Ginestier *et al*, 2007). In established breast cancers, ALDH1 expression has been associated with poor clinical outcome (Ginestier *et al*, 2007) and resistance to chemotherapy (Sladek *et al*, 2002; Tanei *et al*, 2009). Furthermore, studies have indicated that human breast cancers and cell lines contain a sub-population of cells characterised by CD44+/CD24^−/low^/Lin− cell surface markers, and a partial overlap between CD44+/CD24^−/low^/Lin− and ALDH1-positive populations was reported (Al-Hajj *et al*, 2003; Ginestier *et al*, 2007; Fillmore and Kuperwasser, 2008). It is noteworthy that putative cancer stem cells expressing the combined CD44+/CD24^−/low^/ALDH1+ phenotype showed an especially high tumourigenic capacity, being able to form tumours from as few as 20 cells (Ginestier *et al*, 2007).

The importance of BMI-1, a transcriptional repressor of the polycomb group of transcription factors (Alkema *et al*, 1993) and a key regulator of self-renewal in both normal and malignant stem cells (Liu *et al*, 2006), has been more controversial. Still, BMI-1 has been linked to mammary carcinogenesis in some previous studies (Dimri *et al*, 2002; Datta *et al*, 2007). Although some find BMI-1 expression to be associated with a favourable prognosis (Kim *et al*, 2004; Arnes *et al*, 2008; Choi *et al*, 2009), others have reported the opposite (Glinsky *et al*, 2005; Silva *et al*, 2007).

Breast cancers in African populations and among African Americans seem to be more aggressive than breast cancers in Caucasians (Ikpatt *et al*, 2002; Jones *et al*, 2004), and better insight about differences in tumour characteristics (Porter *et al*, 2004; Fregene and Newman, 2005; Morris *et al*, 2007; Bird *et al*, 2008) may suggest strategies to improve clinical management among Africans. In general, there is some evidence that the limitation of chemotherapy and radiation treatment may be associated with the inability to target breast cancer stem cells (Phillips *et al*, 2006; Fillmore and Kuperwasser, 2008; Li *et al*, 2008; Tanei *et al*, 2009), and the efficacy of HER2 inhibitors may relate to their influence on stem cell populations in HER2-positive tumours (Korkaya *et al*, 2008).

On this background, the purpose of our study was to examine the expression of candidate stem cell markers ALDH1 and BMI-1 in breast cancer in relation to basal-like markers, other molecular features and clinicopathological phenotype. These markers were examined in tumours from an African population in which breast cancer is assumed to be more aggressive and also associated with frequent basal-like differentiation (Nalwoga *et al*, 2007). In these populations, early diagnosis and effective treatment is especially challenging (Gakwaya *et al*, 2008).

## Materials and methods

### Patient series

Cases of primary breast carcinoma with available and technically suitable archival paraffin blocks from the period 1990 to 2002 were identified in the Kampala Cancer Registry at the Department of Pathology, Makerere University College of Health Sciences, Kampala (Uganda). The Registry serves an area of about 1914 km^2^, which comprises of Kampala with neighbouring urban and semi-urban areas (Gondos *et al*, 2005) and an estimated population of 1.7 million in 2002 (Population Council, 2004). The population of females >15 years of age is about 530 000. The Baganda from the Central region is the largest ethnic group in the county, but other ethnic groups are represented. The registry methods of collecting data and results have been previously reported (Wabinga *et al*, 1993).

Altogether, 192 cases were included in the study, and 87 other cases with inadequate tissue available were excluded. Clinical information was obtained from the histology reports. The mean age was 46.2 years (range 18–80 years). Duration of symptoms as reported by 127 patients at the time of presentation was 17.1 months on average (median 9 months; range 0.5–108 months). The cutoff point for long duration was 9 months (median value). Stage of disease at the time of diagnosis was available in only 22 patients; the majority (*n*=12) were in stage 4, 8 (36%) were in stage 3, whereas stage 1 and 2 contributed 9%. All cases were re-examined histologically (by HN and JBA) and classified according to the World Health Organisation (Tavassoli and Devilee, 2003) and histological grading was performed in accordance with the Nottingham criteria (Elston and Ellis, 1991). Nuclear grade and mitotic count was also recorded as separate variables according to the same criteria. The permission to conduct this research was obtained from the Research Ethical Committee at Makerere University College of Health Sciences.

### Tissue microarray

The tissue microarray (TMA) technique has been validated in several studies (Camp *et al*, 2000). TMA was performed on 192 cases using archival tissues of invasive breast carcinomas according to Kononen *et al* (1998). Representative tumour areas were identified on haematoxylin and eosin-stained slides, and a minimum of three tissue cylinders (diameter 1 mm) were punched from selected areas of the donor block and mounted into the recipient paraffin block using a custom-made precision instrument (Beecher Instruments, Silver Spring, MD, USA). Sections of 5 *μ*m thickness of the resulting TMA blocks were made by standard technique.

### Immunohistochemistry

The TMA sections were stained with antibodies as shown in Table 1. Sections were deparaffinised in xylene, rehydrated through a series of graded alcohols and rinsed in distilled water. Antigen retrieval based on microwave oven heating with retrieval buffer at 750 W for 10 min followed by 350 W for 15 min (an extra 5 min at 350 W was added for p53, p63 and BMI-1, and 15 min for Ki-67) was used for all antibodies, except epidermal growth factor receptor (EGFR) for which proteinase predigestion for 10 min was applied. Tris–EDTA pH 9.0 retrieval buffer was used for all markers except ALDH1 for which citrate buffer pH 6.0 was used. Sections were allowed to cool at room temperature for 20 min and then thoroughly rinsed in buffer solution and placed in the Dako Autostainer (DakoCytomation, Glostrup, Denmark) for staining. Endogenous peroxidase activity was blocked by incubating sections with 0.03% hydrogen peroxidase containing sodium azide for 5 min, followed by rinsing with buffer solution. Then sections were incubated with specific antibodies at room temperature. Regarding antibodies, P-cadherin and ALDH1 were obtained from BD Biosciences (Oxford, UK), mouse anti-EGFR was obtained from Zymed Laboratories (San Francisco, CA, USA), and BMI-1 was produced as previously described (Arnes *et al*, 2008), whereas all other antibodies were obtained from DakoCytomation A/S. All antigens were detected by the DakoCytomation EnVision+ system-horseradish peroxidase for 30 min, except BMI-1 for which the CSA II kit (DakoCytomation) was used. After rinsing the sections in buffer solution, we developed the peroxidase by incubating with freshly prepared 3,3′-diaminobenzidine chromogen solution for 10 min. Sections were then rinsed in distilled water and counterstained with Meyer's haematoxylin. Cases of breast or colonic carcinoma previously known to be positive for the markers studied were used as positive controls. For c-kit, a gastrointestinal stromal tumour (GIST) was used.

### Evaluation of staining

Tumours without interpretable cores (2.6–4.7%) because of insufficient tumour tissue were omitted from the analysis. A total of 183–187 cases could be evaluated for the various markers. The oestrogen receptor (ER), progesterone receptor (PR), HER2, EGFR, Ki-67, p53, p63, cytokeratin (CK) 5/6, P-cadherin, c-kit and BMI-1 were evaluated as previously described (Engelsen *et al*, 2006; Stefansson *et al*, 2006; Arnes *et al*, 2008; Nalwoga *et al*, 2008). Regarding ALDH1, cytoplasmic staining was evaluated, whereas nuclear staining alone was considered nonspecific and was not included in the analysis.

For BMI-1, a staining index (values 0–9) was determined by multiplying the score for intensity of staining (none=0, weak=1, moderate=2 and strong=3) with the score for proportion of tumour cells stained (<10%=1, 10–50%=2, >50%=3) (Arnes *et al*, 2008). The majority of cases (75%) had staining index 0, and therefore the cutoff was 0=negative and 1–9=positive. For ALDH1, the median staining index was 2, and the cutoff point was set at 0–2=negative and 3–9=positive. Ki-67 proliferative rate was determined as previously described (Nalwoga *et al*, 2007). The cutoff point for Ki-67 was set at 20.0% based on the median value for this series.

### Molecular subtypes

There is no consensus on how to define different molecular subtypes of breast cancer by immunohistochemical markers, and overlapping categories exist. We used criteria on the basis of this literature (Carey *et al*, 2006; Yang *et al*, 2007; Sihto *et al*, 2008) for subclassification into molecular subtypes. In accordance with Carey *et al* (2006), we defined the luminal A (ER+ and/or PR+, HER2−), luminal B (ER+ and/or PR+, HER2+), HER2+ subtype (ER−, PR−, HER2+) and the basal-like subtype (ER−, HER2− and CK 5/6+ and/or EGFR+) subgroups. Tumours negative for all the five markers (ER, PR, HER2, CK 5/6 and EGFR) were considered as unclassified. This definition for luminal B tumours does not identify all luminal B tumours because only 30–50% are HER2+ and the rest are classified with the luminal A. We therefore merged luminal A and luminal B into the luminal subtype. Further, in accordance with our previous studies, we included P-cadherin staining in the definition of BLP (Nalwoga *et al*, 2007; Arnes *et al*, 2008). Using the Arnes *et al* (2008) criteria, we defined BLP profiles as follows: BLP1: concurrent ER−, HER2− and CK 5/6+ BLP2: concurrent ER−, HER2− and P-cadherin+ BLP3: concurrent ER−, HER2− and EGFR+ BLP4: concurrent ER−, HER2− and CK 5/6+ and/or EGFR+ BLP5: concurrent ER−, HER2− and positivity for one or more basal markers (CK 5/6, P-cadherin and EGFR). BLP4 is identical to the core basal phenotype as defined by Nielsen *et al* (2004) and Tischkowitz *et al* (2007).

### Statistical analysis

Statistical analysis was performed using the SPSS version 15.0 software (SPSS Inc, Chicago, IL, USA). We examined the association between ALDH1 and BMI-1 expression with other tumour characteristics using *χ*^2^-test and Fisher's exact test. The *t*-test was used to detect the differences in average age between groups. A *P*-value of <0.05 was considered significant for any statistical test used.

## Results

**Figure 1:**
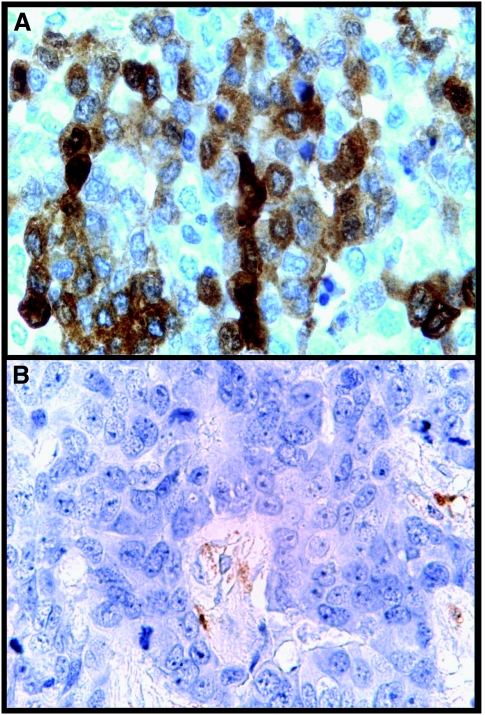
Positive (**A**) and negative (**B**) expression of aldehyde dehydrogenase 1 (ALDH1) protein in tumour cells of breast carcinomas (both × 400 magnification).

In all, 88 tumours (48%) were positive for ALDH1, whereas 95 (52%) were negative for ALDH1 (Figure 1). The majority (62%) of ALDH1-positive cases were high-grade ductal carcinomas. Altogether, 40 cases (46%) showed staining in >10% of the tumour cells, whereas 16 of 88 (18%) cases had a diffuse staining in ⩾50% of the tumour cells. Overall, the expression of ALDH1 seemed to be evenly distributed throughout the tumour cell population, although there were some cases with clusters of positively stained cells within the diffuse pattern. The average percentage of stained tumour cells in positive cases was 18%. Of the ALDH1-positive tumours, 31% were of the luminal subtype (27.3% luminal A, 3.4% luminal B), 31% had a basal-like subtype (core basal phenotype; BLP4), 16% were in the HER2 subtype and 23% were in the unclassified category. A majority (53%) of the ALDH1-positive cases were triple-negative tumours.

Table 2 shows ALDH1 expression and associations with clinicopathological characteristics. Patients with a shorter duration of symptoms were more likely to express ALDH1 than those with longer duration of symptoms (odds ratio 2.2; 95% confidence interval 1.05–4.5, *P*=0.036). The ALDH1 expression was significantly associated with markers of poor prognosis, such as high histological grade, high mitotic counts, high nuclear grade, ER negativity, PR negativity, and p53 expression. No associations were found between ALDH1 expression and HER2 status, p63 or c-kit positivity.

As shown in Table 3, CK5/6 was positive in 15%, P-cadherin in 27% and EGFR in 20% of all cases. One or more of these were positive in 33% of the cases (61 of 185). A total of 86 tumours (46%) were of the luminal subtypes (42%, luminal A, 4% luminal B), 22% (41 of 186) had a basal-like subtype, the HER2 subtype contributed 12% (23 of 186), and 19% (36 of 186) were in the unclassified group. Regarding the different BLP profiles, 15% (27 of 186) were BLP1, 22% (41 of 187) were BLP2, 17% (31 of 186) were BLP3, 22% (41 of 186) were BLP4 (core basal phenotype) and 26% (49 of 186) were BLP5. All tumours in the different BLP profiles were triple negative in this series. A majority of the triple-negative tumours showed basal-like differentiation; 53% (41 of 77) had a core basal profile (BLP4), whereas 64% (49 of 77) of the TNP tumours had positive expression of at least one of the three basal markers (CK5/6, P-cadherin, EGFR) combined with ER– and HER2–, corresponding to the BLP5 profile.

Table 3 also shows the relationship between ALDH1 positivity and molecular subtypes of breast cancer. The ALDH1 expression was significantly associated with molecular subtype and BLP profiles as defined in this paper, as well as with TNP and individual basal markers CK 5/6 and EGFR. Thus, the BLP, the HER2 subgroup and the unclassified category were more likely to express ALDH1 than the luminal subtypes.

In all, 46 tumours (25%) were positive for BMI-1 staining. The majority of cases (61%) were of the luminal subtype (54.3% luminal A, 6.5% luminal B), whereas the basal-like category contributed 22%, 11% were in the HER2 subgroup and 7% were unclassified. In total, 13 tumours (28%) were triple negative. The BMI-1 positivity was mostly associated with features of good prognosis, such as low histological grade (*P*=0.011), low mitotic counts (*P*=0.010) and ER positivity (*P*=0.001). Further, BMI-1 expression was inversely associated with the TNP (*P*=0.037) and with ALDH1 positivity (*P*=0.009). Tumours in the luminal subtype (odds ratio 5.4; 95% confidence interval 1.05–19.2, *P*=0.005) were more likely to express BMI-1 than unclassified tumours. No association was found between BMI-1 expression and the other subtypes, the basal markers such as CK5/6, P-cadherin, EGFR, and the BLP profiles.

## Discussion

In this study, our aim was to explore the expression of candidate stem cell markers ALDH1 and BMI-1 in breast cancers from an African population and their possible associations with BLP and other molecular markers. We found that ALDH1 expression was associated with features of aggressive tumours such as high histological grade, high nuclear grade, high mitotic count, p53 expression and ER/PR negativity. In addition, ALDH1 expression was associated with a short duration of symptoms. Thus, ALDH1 status might represent an indicator of aggressive breast cancer (Ginestier *et al*, 2007; Morimoto *et al*, 2009). In support of this, others have suggested that the amount of cancer stem cells within breast tumours may correspond to the risk of distant metastases (Abraham *et al*, 2005; Glinsky *et al*, 2005).

It has been observed that basal-like breast cancers might be enriched with CD44+/CD24− cells (Honeth *et al*, 2008), and an overlap between CD44+/CD24− cells and ALDH1-positive cell populations were described (Ginestier *et al*, 2007). Moreover, the CD44+/CD24−/ALDH1+ phenotype identified a highly tumourigenic cell population that was able to form tumours from as few as 20 cells. Our results showed that ALDH1 was significantly associated with the basal-like subtype and different BLP profiles, as well as with individual basal markers CK 5/6 and EGFR, similar to what others have reported (Ginestier *et al*, 2007). To speculate, our findings might be related to the aggressive behaviour and therapy resistant features of the basal-like breast cancer subtype (Sorlie *et al*, 2001; Banerjee *et al*, 2006; Fillmore and Kuperwasser, 2008; Li *et al*, 2008). Moreover, we found a significant association between ALDH1 expression and the triple-negative tumours, a group whose poor prognosis has been widely reported (Dent *et al*, 2007).

Our findings indicate a higher frequency of ALDH1 expression (48%) in this series of breast cancer from an African population, compared with 19 and 30% in two different Caucasian populations described by Ginestier *et al* (2007). We also found more extensive staining in positive cases (Ginestier *et al*, 2007). Further, in comparison with data derived from breast tumours in Caucasian and Asian populations (Ginestier *et al*, 2007; Morimoto *et al*, 2009; Tanei *et al*, 2009) regarding ALDH1 positivity rate in tumours with similar characteristics (histological grade, ER, HER2, Ki-67), we observed that tumours from our present series stained in a higher percentage of cases in most poor prognosis categories (such as high histological grade, ER-negative cases, HER2-negative cases, tumours with high Ki-67 expression). Hence, apart from methodological discrepancies, biological differences might be present when comparing breast cancers from African and Caucasian populations (Elledge *et al*, 1994; Ikpatt *et al*, 2002; Jones *et al*, 2004; Porter *et al*, 2004). In line with this, a poorer outcome has been observed in African and African-American patients (Wojcik *et al*, 1998; Ikpatt *et al*, 2002) when compared with breast cancers among Caucasians, with differences in the spectrum of tumour characteristics and prognostic features such as the presence of tumour necrosis, low ER positivity rate, high HER2-positive rate, and a high frequency of basal-like features (Mbonde *et al*, 2001; Ikpatt *et al*, 2002; Carey *et al*, 2006; Nalwoga *et al*, 2006, 2007; Morris *et al*, 2007; Bird *et al*, 2008).

In contrast to our findings on ALDH1, the expression of BMI-1, another candidate stem cell marker (Arnes *et al*, 2008), was inversely associated with ALDH1 and related to features of good prognosis, such as low histological grade, low mitotic count, ER positivity and absence of TNP (Kim *et al*, 2004; Choi *et al*, 2009). This is in line with our recent studies of breast cancer (Arnes *et al*, 2008) and other tumours (Bachmann *et al*, 2008; Engelsen *et al*, 2008). The frequency of BMI-1 expression (25%) was lower than those found in other studies (43–62%) (Kim *et al*, 2004; Arnes *et al*, 2008; Choi *et al*, 2009). Others have found different results, BMI-1 expression being associated with more aggressive tumours (Glinsky *et al*, 2005; Silva *et al*, 2007). In addition, Glinsky *et al* (2005) found that expression of a BMI-1-driven 11 gene signature was associated with risk of metastases in breast carcinoma. The explanation for this inverse relationship is not known.

In conclusion, we observed a high prevalence of ALDH1 staining in this series of invasive breast carcinomas from Uganda. Expression of ALDH1 was significantly associated with a BLP and with features of aggressive tumours. Assessment of ALDH1 expression might help to identify a high-risk (Sreerama and Sladek, 1997) subgroup of breast cancers in this population. More studies are required to elucidate the possible significance of these stem cell markers in breast cancer patients.

## Figures and Tables

**Table 1:** Immunohistochemical staining procedures

**Biomarker**	**Antibody**	**Clone**	**Dilution**	**Incubation time (min)**
ER	MMA oestrogen receptor-*α*	1D5	1 : 50	30
PR	MMA progesterone receptor	PgR 636	1 : 150	30
HER2	RA c-*erb*B-2 oncoprotein	Polyclonal	1 : 500	60
Ki-67	MMA Ki-67 antigen	MIB-1	1 : 50	60
p53	MMA p53 protein	DO-7	1 : 1000	60
p63	MMA p63 protein	A4A	1 : 300	30
CK 5/6	MMA cytokeratin 5/6	D5/16 B4	1 : 200	30
P-cadherin	P-cadherin purified MA	56	1 : 400	60
EGFR	Mouse anti-EGFR	31G7	1 : 30	30
c-kit	RA CD117, c-kit	Polyclonal	1 : 200	30
ALDH1	Purified M anti-ALDH	44	1 : 250	60
BMI-1	Anti-BMI-1	6C9	1 : 1	60

Abbreviations: MMA=monoclonal mouse antihuman; MA=mouse antihuman; RA=rabbit antihuman; M=mouse; ER=oestrogen receptor; PR=progesterone receptor; EGFR=epidermal growth factor receptor; ALDH1=aldehyde dehydrogenase 1; CK=cytokeratin.

**Table 2:** ALDH1 expression and associations with clinicopathological features and molecular characteristics

**Variable**	**ALDH1 negative (*n*; %); *n*=95**	**ALDH1 positive (*n*; %); *n*=88**	**OR (95% CI)**	***P*-value**
*Age in years*				
<50	52 (52)	48 (48)	1.0	
⩾50	37 (49)	38 (51)	1.1 (0.6–2.0)	NS
				
*Duration of symptoms*				
⩽9 months	26 (41)	37 (59)	1.0	
>9 months	35 (60)	23 (40)	0.5 (0.2–1.0)	0.036
				
*Histological type*				
Ductal	82 (51)	79 (49)	1.0	
Others	13 (62)	8 (38)	1.6 (0.6–4.0)	NS
				
*Histological grade*				
Grade 1	20 (87)	3 (13)	1.0	
Grade 2	38 (59)	26 (41)	4.6 (1.2–16.9)	0.016
Grade 3	37 (39)	58 (61)	10.5 (2.9–37.6)	0.000
				
*Nuclear grade*				
Grade 1	18 (72)	7 (28)	1.0	
Grade 2	48 (63)	28 (37)	1.5 (0.6–4.0)	NS
Grade 3	29 (36)	52 (64)	4.6 (1.7–12.3)	0.001
				
*Mitotic count*				
0–6	31 (71)	13 (29)	1.0	
7–13	29 (67)	14 (33)	1.5 (0.5–2.9)	NS
>13	35 (37)	60 (63)	3.3 (1.9–8.8)	0.000
				
*Ki-67*				
Low (<20%)	50 (57)	38 (43)	1.0	
High (⩾20%)	45 (47)	50 (53)	1.4 (0.8–2.6)	NS
				
*p53*				
Low SI (0–4)	73 (57)	55 (43)	1.0	
High SI (>4)	22 (40)	33 (60)	2.0 (1.05–4.8)	0.034
				
*ER*				
Positive	48 (69)	22 (31)	1.0	
Negative	47 (42)	66 (58)	3.1 (1.6–5.7)	0.000
				
*PR*				
Positive	35 (67)	17 (33)	1.0	
Negative	60 (46)	71 (54)	2.4 (1.2–4.7)	0.009
				
*HER2*				
Negative	82 (54)	71 (46)	1.0	
Positive	13 (43)	17 (57)	1.5 (0.7–3.3)	NS

Abbreviations: ALDH1=aldehyde dehydrogenase 1; OR=odds ratio; CI=confidence interval; NS=not significant; SI=staining index; ER=oestrogen receptor; PR=progesterone receptor.

**Table 3:** ALDH1 expression and associations with basal markers, molecular subtypes, BMI-1, c-kit and p63 expression

**Variable**	**ALDH1 negative (*n*; %); *n*=95**	**ALDH1 positive (*n*; %); *n*=88**	**OR (95% CI)**	***P*-value**
*CK 5/6*				
Negative	86 (56)	68 (44)	1.0	
Positive	8 (29)	20 (71)	3.2 (1.3–7.6)	0.008
				
*P-cadherin*				
Negative	75 (56)	58 (44)	1.0	
Positive	20 (40)	30 (60)	1.9 (1.0–3.8)	0.048
				
*EGFR*				
Negative	81 (56)	64 (44)	1.0	
Positive	12 (33)	24 (67)	2.5 (1.1–5.4)	0.015
				
*BLP1*				
Absent	86 (56)	69 (44)	1.0	
Present	8 (30)	19 (70)	2.9 (1.2–7.4)	0.013
				
*BLP2*				
Absent	80 (56)	62 (44)	1.0	
Present	15 (37)	26 (63)	2.2 (1.1–4.6)	0.026
				
*BLP3*				
Absent	84 (56)	67 (44)	1.0	
Present	10 (32)	21 (68)	2.6 (1.2–6.0)	0.018
				
*BLP4 (CBP)*				
Absent	80 (57)	61 (43)	1	
Present	14 (34)	27 (66)	2.5 (1.2–5.2)	0.011
				
*BLP5*				
Absent	77 (58)	56 (42)	1.0	
Present	17 (35)	32 (65)	2.6 (1.3–5.1)	0.005
				
*TNP*				
No	65 (61)	41 (39)	1.0	
Yes	30 (39)	47 (61)	2.5 (1.4–4.5)	0.003
				
*Molecular subtype*				
Luminal	56 (68)	27 (32)	1.0	
HER2	8 (36)	14 (64)	3.6 (1.4–9.7)	0.008
Basal-like	14 (34)	27 (66)	4.0 (1.8–8.8)	0.000
Unclassified	16 (44)	20 (56)	2.6 (1.2–5.8)	0.018
				
*c-kit*				
Negative	91 (52)	84 (48)	1.0	
Positive	4 (50)	4 (50)	1.1 (0.3–4.4)	NS
				
*p63*				
Negative	80 (53)	71 (47)	1.0	
Positive	15 (47)	17 (53)	1.3 (0.6–2.7)	NS
				
*BMI-1*				
Negative	64 (46)	74 (54)	1.0	
Positive	31 (69)	14 (31)	0.4 (0.2–0.8)	0.009

Abbreviations: ALDH1=aldehyde dehydrogenase 1; OR=odds ratio; CI=confidence interval; NS=not significant; EGFR=epidermal growth factor receptor; CK=cytokeratin; CBP=core basal phenotype; TNP=triple-negative phenotype.
